# Functional analysis of telomere maintenance mechanisms is more informative than immunohistochemistry for *ATRX* mutation interpretation in Gliomas

**DOI:** 10.1186/s40478-025-02164-z

**Published:** 2025-12-20

**Authors:** Clemence Guerriau, Camille Léonce, Catherine Carpentier, Karima Mokhtari, Franck Bielle, Amel Dridi-Aloulou, Patrick Lomonte, David Meyronet, Marc Sanson, Luis Castro-Vega, Delphine Aude Poncet

**Affiliations:** 1 Institut de Pathologie Est, Hospices Civils de Lyon, Univ Lyon, Université Claude Bernard Lyon 1, Lyon, France; 2 CNRS UMR 5261, INSERM U1315, LabEx DEV2CAN, Institut NeuroMyoGène-Pathophysiology and Genetics of Neuron and Muscle (INMG-PGNM), Univ Lyon, Université Claude Bernard Lyon 1, Lyon, France; 3 INSERM 1052, CNRS 5286, Centre Léon Bérard, Cancer Research Center of Lyon, Univ Lyon, Claude Bernard Lyon 1 University, Lyon, France; 4 Paris Brain Institute (ICM), Hôpital Pitié-Salpêtrière, Inserm U 1127, CNRS UMR 7225, Genetics and Development of Brain Tumors Team, Sorbonne University, Paris, France; 5 AP-HP, Hôpital de la Pitié-Salpêtrière-Charles Foix, Service de Neurologie 2-Mazarin, Hôpital de La Pitié Salpêtrière, 75013 Paris, France; 6 Département de Neuropathologie, Laboratoire Escourolle, Hôpital de La Pitié-Salpêtrière, AP-HP, 75013 Paris, France

**Keywords:** Glioblastoma, IDH-mutant, ATRX, ALT, Telomere, Database, Telomerase, MAPK, Extend

## Abstract

Loss of ATRX function, a diagnostic criterion of IDH-mutant astrocytoma, is closely associated with alternative lengthening of telomeres (ALT), a telomere maintenance mechanism (TMM). As immunohistochemical (IHC) assessment of ATRX is error-prone, sequencing has been integrated into clinical workflows. While frameshift and nonsense variants can be classified as loss-of-function (LOF) mutations, missense variants remain difficult to interpret. To address this, we analyzed *ATRX*-altered gliomas from TCGA (N = 539 tumors, 587 alterations) and a local cohort of 100 diffuse gliomas. Aside from IDH-mutant astrocytoma and H3.3-mutant glioma, glioblastoma (13–19%), oligodendroglioma (2%), and MAPK-altered tumors (2–7%) were consistently represented in both cohorts. Missense mutations accounted for 18% (TCGA) and 28% (local) of variants. Functional annotation of TCGA missense mutations using IHC, NGS-based TMM status, transcript levels, and RNA-seq–derived telomerase signatures (EXTEND) allowed us to functionally annotate only 3 of 106 variants. Therefore, we directly assessed the TMM status in the local cohort, using the TeloDIAG assay and retrieved 80% of ALT-positive samples, corresponding to 96% (N = 53) of IDH-mutant astrocytomas and 74% (N = 19) of histone-mutant gliomas, as expected. Importantly, ALT activity was also observed in 42% (N = 19) of glioblastomas and 86% (N = 7) of MAPK-altered gliomas, indicating potential implications for clinical behavior and therapeutic targeting. While sensitivity for LOF variant detection was similar—88% for TMM and 80% for IHC—TMM performed better for missense alterations (85 vs. 65%) and is more discriminant in overall survival prediction. Overall, 83% (N = 24) of missense mutations associated with an ALT phenotype were located within the major functional domains of the ATRX protein, compared to only 59% when considering all missense mutations, highlighting the functional relevance of the assay. Collectively, these findings emphasize the clinical value of functional TMM assessment in refining glioma diagnosis. Last, targeting of ALT-associated DNA-damage deficiency and immunogenic context is under clinical investigation, highlighting the promising theranostic potential of TMM assessment.

**Supplementary Information:**

The online version contains supplementary material available at 10.1186/s40478-025-02164-z.

## Introduction

*ATRX* stands for X-linked alpha-thalassemia/mental retardation syndrome, due to its initial identification in patients with severe psychomotor retardation, characteristic facial features, genital abnormalities, and alpha-thalassemia [1]. ATRX exhibits ATP-dependent nucleosome remodeling activity and is specialized in depositing the histone variant H3.3 at repetitive, transcriptionally inactive sites, such as pericentromeric and telomeric heterochromatin [2]. ATRX also contributes to telomere maintenance and replication, partly by regulating the formation and/or resolution of G-quadruplex structures (intramolecular rearrangements of the telomeric G-rich strand) [3] and R-loops [4] (RNA: DNA heteroduplexes). Hence, ATRX loss induces replicative stress at telomeres [3], and its inactivation facilitates the initiation of the “alternative lengthening of telomere” (ALT) process [5, 6]. ALT is an “alternative” telomere maintenance mechanism (TMM) to telomerase reactivation, used by 7–10% [7] of tumors to maintain telomere length and avoid senescence or mitotic crisis. This process relies on aberrant repair mechanism, produces extrachromosomal telomere sequences (ECTR), mainly occurs in ALT-associated PML bodies (APBs), and results in long heterogeneous telomeres (for review see [8]). Thus, ALT can be detected on the basis of telomere length by Telo-FISH [7], Telo-qPCR [9] or long read sequencing [10]. The latter two technologies also quantify ECTR, such as C-circle (CC), which are partially double-stranded telomeric circle detected after a pre-amplification step [11].

In humans, neuroepithelial and mesenchymal tumors, such as gliomas and sarcomas, show the highest rates of ALT activation [12, 13]. Importantly, in gliomas, ATRX loss (detected by immunohistochemistry) is a diagnostic criterion for grade 2 to 4 IDH-mutant astrocytomas, which represent 20–25% of diffuse gliomas [14]. ATRX loss is also frequently observed in H3.3-mutated gliomas (G34R/V and K27M), including nearly 100% of hemispheric G34R/V-mutant gliomas and approximately 30% of midline diffuse gliomas [15]. ATRX loss is often associated with *TP53* loss and genomic instability [16, 3]. Other diffuse gliomas, such as glioblastomas (GBM, 65% of gliomas) and IDH1/2-mutant oligodendrogliomas (OD, 10% of gliomas), typically maintain their telomeres by reactivating the telomerase enzyme through mutations in *TERT* (telomerase reverse transcriptase) promoter (p*TERT*mt), which leads to TERT re-expression [14]. 

 Routine detection of ATRX loss by immunohistochemistry (IHC) faces two significant challenges: (i) inter-observer variability, and (ii) inability to detect mutations that do not affect protein stability, resulting in 11 to 21% false-negative results compared to the Telo-FISH method [17, 18, 15]. Sequencing of the *ATRX* gene, which spans approximately 300 kb and includes 36 exons, has recently become an alternative due to the widespread adoption of next-generation sequencing (NGS). However, interpreting the clinical significance of mutations remains challenging in the absence of a functional database for *ATRX*. While hemi/homozygous deletion, frameshift and re-arrangement are considered as loss-of-function (LOF) and “probably pathogenic” alterations, the impact of missense mutations and small in-frame insertions/deletions remains unclear.

We therefore propose (i) to exhaustively describe the spectrum of *ATRX* alterations in a large cohort of 591 gliomas from the TCGA, in relation to published data on TMM, and (ii) to functionally assess the ALT process in a cohort of 100 gliomas, correlating findings with standard clinical diagnostic criteria (NGS, IHC). Importantly, these cohorts include gliomas beyond astrocytomas, covering the full spectrum of contexts in which *ATRX* mutations occur. For functional testing, we employed the TeloDIAG, a combined assay that integrates telomere content quantification [19] with CC detection [11], thereby providing a robust evaluation of the functional consequences of *ATRX* mutations for clinical glioma diagnosis.

## Material and methods

### Design and setting of the study

For the first dataset, clinical and histomolecular information was extracted from the cBioPortal interface of the cancer Genome Atlas (TCGA). A total of 12 studies were selected: brain_cptac_2020, difg_glass_2019, gbm_columbia_2019, gbm_cptac_2021, gbm_mayo_pdx_sarkaria_2019, gbm_tcga, glioma_msk_2018, glioma_mskcc_2019, lgg_ucsf_2014, lgggbm_tcga_pub, odg_msk_2017, and past_dkfz_heidelberg_2013. ATRX immunohistochemistry analyses were performed at contributing institutions with their own local methods. After removing duplicates (on the basis of sample ID), 3,924 cases were compiled. Filtering for *ATRX* alterations resulted in the selection of 540 samples, encompassing 591 genetic alterations. The sequencing methods reported in Supplementary Table 1 included targeted exome sequencing (e.g., FoundationOne T5/T7, MSK-IMPACT 341/410/468, or in-house WES) for 297 tumors and whole-genome sequencing (WGS) for 12 tumors; information is missing for 281 samples. The allelic variant frequency (VAF) of *ATRX* mutations, ranging from 0.03 to 0.99, was compared to the VAF of other alterations within the same sample and classified as either *heterozygous (wild-type allele present) or **homozygous/hemizygous (no wild-type allele) (Supplementary Table 1). Frameshift, nonsense, splicing-site mutations and gene rearrangements were classified as loss-of-function (LOF) alterations. Diagnoses were assigned according to the WHO 2021 classification, based on available histological and molecular parameters.

The second dataset consists of 100 gliomas from the Pitié Salpétrière Onconeurotek collection (certified 96900), which had previously undergone *ATRX* sequencing for diagnostic purposes. ATRX labelling was done as previously published [20]. Briefly, FFPE sections. (3 μm thick) were deparaffinized and immunolabelled with a Ventana Benchmark XT stainer (Roche, Basel, Switzerland), using the mouse monoclonal anti-ATRX (Bio SB, clone BSB-108, BSB3296, 1:100). The loss of expression was defined as the total absence of nuclear labelling in tumor cells associated with a maintained expression in normal cells (positive internal control) within the same tissue area. All the patients signed a written informed consent (CPP authorization N° 2023-A02763-42). The recommended time between surgery and specimen preservation was 2 h for this collection; 89 samples were Formalin-Fixed Paraffin-Embedded (FFPE) and 11 were frozen. These samples were included for functional testing. Pathogenicity data for missense mutations were available for only 4 variants in ClinVar database. Figures were done using excel and powerpoint (Microsoft).

### ALT status determination

Two telomere parameters were assessed using the TeloDIAG assay, as previously described [21]: telomere content, corresponding to telomere length (TL), and C-circles (CC). TL was quantified by qPCR relative to the reference gene (*RPLP0*) using the formula (TL = E^−CT^_TEL_/E^−CT^_RPLP0_). C-circles, if present, were amplified by rolling circle amplification using the Φ29 DNA polymerase. A sample was considered positive for CC if the ratio of telomeric sequences quantification with and without incubation with the Φ29 (TL_+Φ_/TL_-Φ_) was ≥ 1.3 for FFPE samples and ≥ 1.5 for frozen samples. In regard with our previous publication, samples were considered ALT + in two situations: if CC were detectable or in situation of long telomeres (TL > 1.7 arbitrary units [au]) with or without detectable CC (fixative-induced DNA oxidation and breakage in old samples can open circles and preclude amplification, as previously demonstrated [21]). Samples negative for CC and with short telomeres (TL < 1.7au) were considered to maintain telomeres via telomerase activity.

### Statistical analysis

Statistical analyses and boxplots were generated using R software (R Core Team (2024). *R: A language and environment for statistical computing*. R Foundation for Statistical Computing, Vienna, Austria). Comparisons of group means were conducted using the non-parametric Wilcoxon rank-sum test, survival curves were plotted, using the *survfit* package.

## Results

### Information on *ATRX* alterations in public datasets

**Fig. 1:**
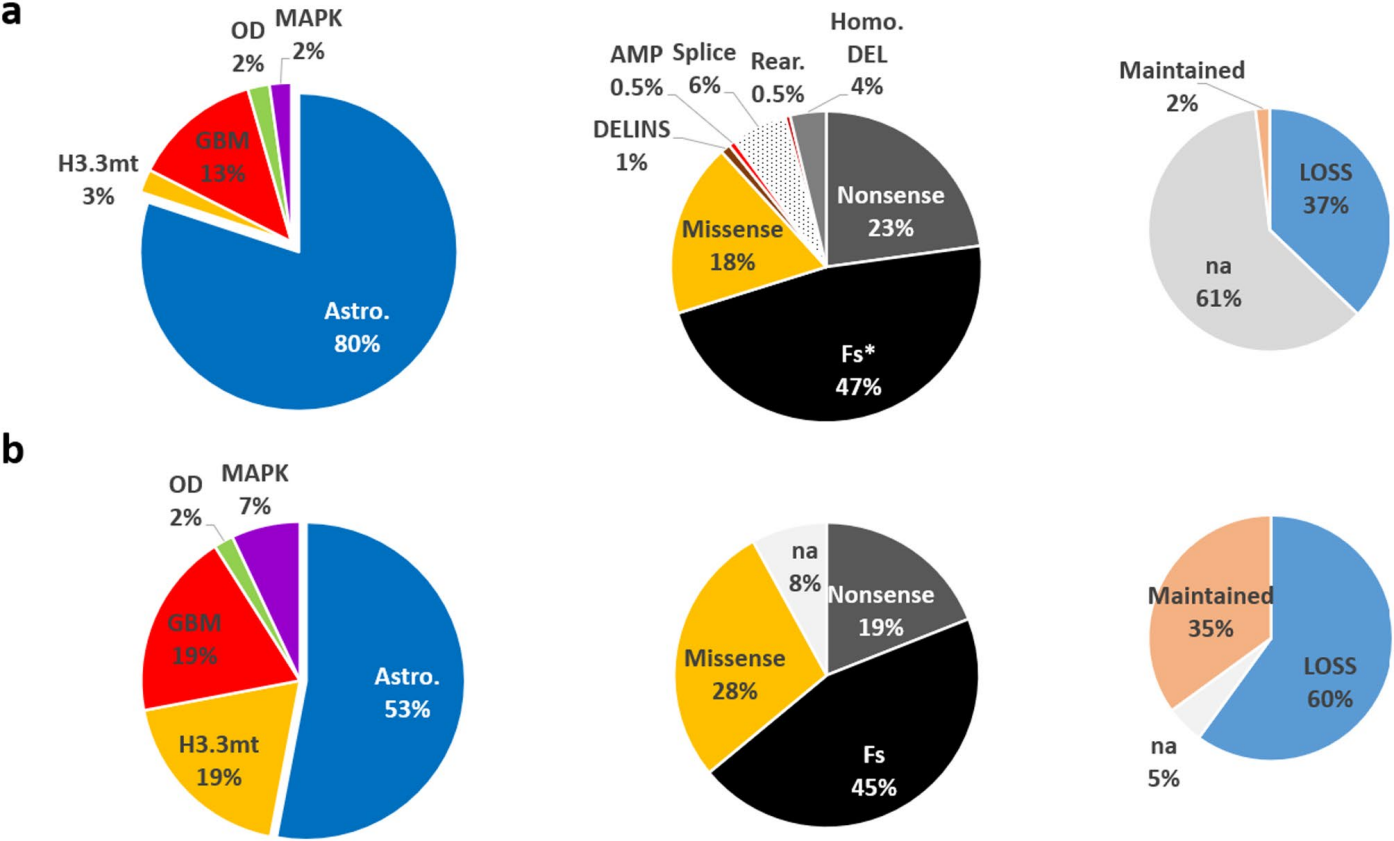
Histomolecular Characterization of the Two Cohorts. Distribution of glioma subtypes (left), types of *ATRX* gene alterations (middle), and ATRX immunolabeling status (right) in the selected TCGA cohorts (N = 587 *ATRX* alterations corresponding to 539 tumors) (**a**) and in our local dataset (N = 100 tumors with 93 *ATRX* alterations) (**b**). GBM, glioblastoma; H3.3, histone H3.3-mutant glioma; MAPK mutant, gliomas with NF1, BRAF, or FGFR1 mutations; OD, oligodendroglioma; Fs, frameshift; DELINS, deletion and/or insertion; AMP, amplification; HOMO DEL, homozygous or hemizygous deletion; na, not available

To evaluate the informativeness of clinical testing, we aggregated data from 12 glioma-focused studies available in the TCGA database. After removing duplicates and filtering by diagnosis according to the WHO 2021 criteria, we selected 539 glioma samples with *ATRX* variants identified by NGS. The majority of gliomas were astrocytomas (80%), but the dataset also included glioblastomas (GBM, 13%) and oligodendrogliomas (OD, 2%), which are typically p*TERT*mt telomerase-positive (TA +) tumors (Fig. 1a). These 539 samples contained a total of 587 *ATRX* variants, as 52 tumors exhibited multiple *ATRX* alterations. The types of alterations included hemi- or homozygous deletions (4%), nonsense mutations (23%), frameshift mutations (47%), missense mutations (18%), splice site mutations (6%), and rare deletions-insertions or rearrangements (Fig. 1a). Except for missense mutations and small INDELs, all these alterations can be classified as loss-of-function (LOF) variants. To further analyze missense mutations, we used immunohistochemistry (IHC) ATRX status, which was available for 218 tumors (40%). Of note, only 2% (N = 11) of these samples retained ATRX expression (ATRXwt), including 10 out of the 11 cases with hemi- or homozygous gene deletions, which are typically associated with protein loss. Similarly, all samples bearing missense mutations (N = 32) were classified as lost for ATRX protein (ATRXloss), despite the expectation that the protein should still be expressed, albeit non-functional. This suggests potential biases in copy number variation (CNV) detection and/or misinterpretation of IHC results. Indeed, all CNVs were detected by targeted exome sequencing, and none by WGS, which may favor misdetection. In addition, high interoperator variability has been reported for ATRX IHC testing compared to functional testing by Telo-FISH [17, 18, 15, 22]. Therefore, IHC data were further ultimately considered non-informative for this analysis.

**Fig. 2:**
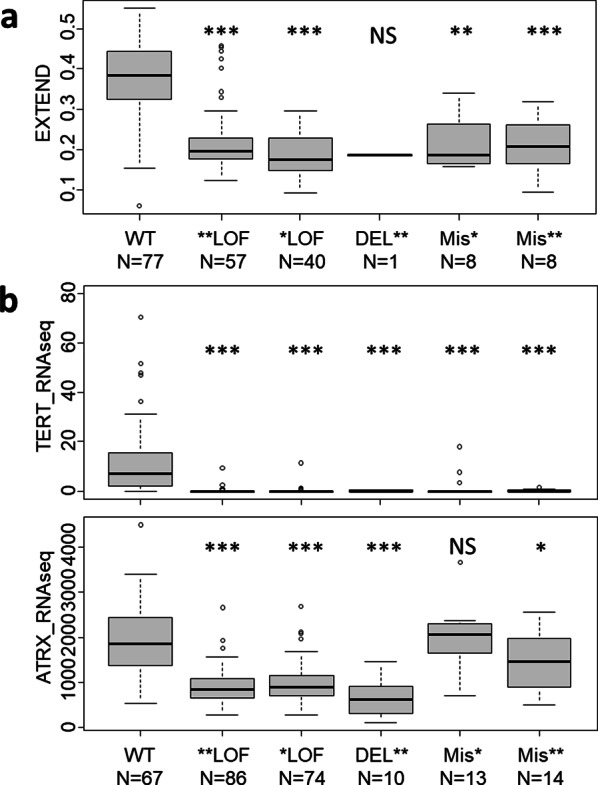
TMM-related Information in the TCGA cohort. The EXTEND signature (N = 191), as well as the expression levels of *ATRX* and *TERT* (N = 264) were retrieved for the TCGA cohort. The control wild-type (WT) samples are IDHwt GBM. WT, wild type; LOF, loss-of-function; DEL, homo/hemizygous deletion; Mis-misense; *—one hit; ** two hits; *p*-value: NS—non significant; * < 0.01; ** < 0.001; *** < 0.0001

Several publications have developed algorithms to predict ALT or TA based on whole-genome sequencing (WGS) [23–26]. We examined these predictions for common TCGA samples. However, these models have almost exclusively included LOF alterations, such as frameshift mutations, that were mainly used as controls to validate the models. As a result, no additional relevant information was retrieved except for two missense mutations, Y2418N and E2172A, classified as ALT- (Supplementary Table 1). Alternatively, data from the EXTEND signature [14], based on RNA-seq analysis and estimating the telomerase activity spectrum, was available for 114 out of the 529 *ATRX*-altered samples. Additionally, we included 77 ATRXwt IDHwt GBM samples as controls. The EXTEND signature aligned with the classification of tumors bearing *ATRX* alterations, all of which belonging to the ALT group (Fig. 2a). However, since the EXTEND signature was developed by comparing p*TERT*mt samples (OD and GBM) versus ATRX/DAXX altered samples (mostly astrocytomas), it may reflect tumor cell origin/entity rather than TMM status, as demonstrated by Nonneville et al. [25]. We then inquired the expression levels of *ATRX* and *TERT* using RNA-seq data available from 197 samples. *TERT* expression was nearly absent in most tumors harboring *ATRX* variants. Interestingly, three single missense variants (V128A, Y2418N, R1131G), found in OD and GBM, were associated with *TERT* expression and preserved ATRX expression; among these, Y2418N was notable. Regarding *ATRX* expression levels, LOF variants showed a clear decrease (Fig. 2b), whereas single missense variants exhibited no reduction compared to WT samples, consistent with the fact that amino-acid changes does not necessarily lead to protein loss. Overall, of the 106 missense mutations analyzed, only four could be classified as “probably benign” or “benign”, and no additional meaningful insight into the TMM could be derived from the other samples.

In summary, we identified 117 missense mutations across 93 samples: 71 samples harbored a single hit, 9 samples had two missense mutations (N = 18), and 13 samples carried at least another LOF alteration. All variants were classified as “non informative”, except for two classed as benign (Y2418N and E2172A) and two as probably benign (V128A, R1131G) [24]. These findings highlight the limitations of NGS-based predictive models and emphasize the need for functional studies to improve the annotation of clinical databases for molecular diagnosis in glioma.

### Functional testing of ALT status in *ATRX*-altered gliomas

As emphasized by Nonneville et al., the CC assay should be used to accurately assess the TMM [25]. We have thus investigated directly the TMM activated by tumors in a cohort of 100 gliomas from the Pitié-Salpêtrière Tumor bank, including 92 with *ATRX* mutations (and 8 histone-mutant without NGS information). This cohort comprised 53 IDH1/2-mutant astrocytomas, 19 histone-mutant tumors, 7 with MAPK pathway alterations (BRAF, NF1), 19 GBM (13 p*TERT*mt) and 2 OD (1p*TERT*mt). Frameshift, nonsense and missense mutations accounted for 45%, 19% and 28% of all mutations, respectively, consistent with the proportions observed in the TCGA cohort (Fig. 1b)(rearrangements and copy number alterations were not addresses in this study). Regarding ATRX status determined by IHC (N = 95), protein expression was lost in 63% (60/95) of tested samples (Fig. 1b). In addition, 20 samples (10 OD and 10 GBM) with p*TERT* mutation and no *ATRX* alteration (NGS and IHC) were used as controls.

**Fig. 3:**
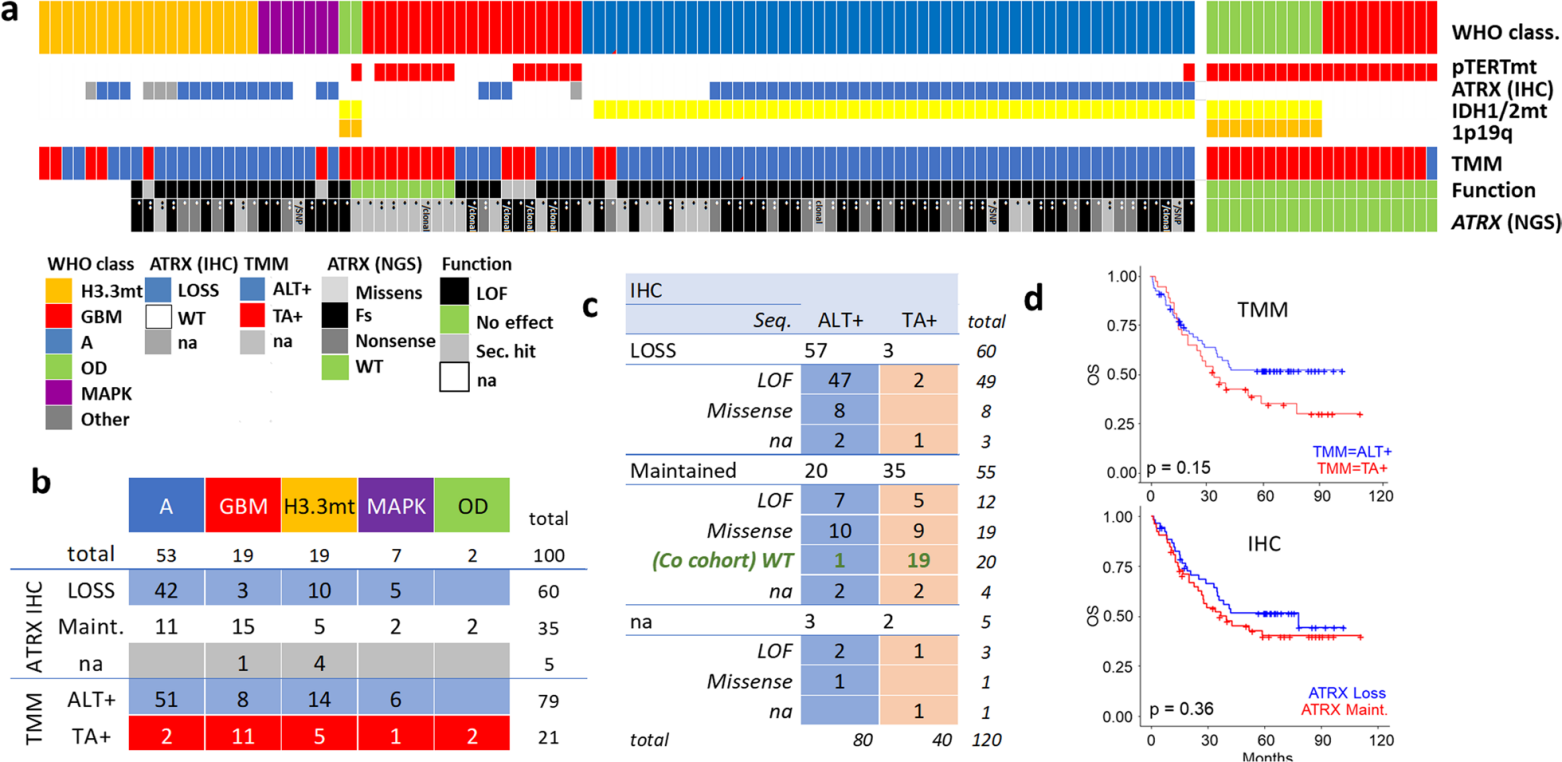
Functional TMM Testing in *ATRX*-Altered Gliomas and control samples. **a** TMM classification as a function of histomolecular diagnostic markers (IDH1/2 mutations, *pTERT* mutations, 1p/19q codeletion), ATRX protein loss (IHC), or *ATRX* gene mutations (NGS). **b** distribution of TMM and ATRX (IHC) status according to glioma subtype in the 100 samples. **c** Functional TMM results (ALT + or TA +) as a function of ATRX immunolabelling status for LOF alterations (N = 64) and missense mutations (N = 28) (N = 8 na values). Results of the control cohort, selected on the criterion of maintained ATRX and no genic alteration, are indicated in green. d) Survival plot are represented for patients with available IHC and TMM values (N = 111). WT, wild type; *one residual allele; **no WT allele; SNP, single nucleotide polymorphism; clonal alteration; na, not available

The TeloDIAG assay [21] assesses the presence of CC and TL to identify ALT + samples; its ALT- counterparts was defined as TA + . All but one control sample (N = 20) were classified as TA + (Fig. 3a). The false positive showed Telo-qPCR cycle thresholds consistent with loss of the reference gene rather than increased telomere content. This situation was not observed in other samples. Considering the 100 samples, 79% were classified as ALT + (Fig. 3a,b). Regarding diagnosis, 96% (51/53) of astrocytomas were classified as ALT + (42 of which showed ATRX loss), along with 73% (14/19) of histone-mutant tumors, consistent with previous studies [15]. Interestingly, among tumors usually assumed to re-express the telomerase, 42% (8/19) of GBMs with *ATRX* alteration were indeed ALT + (2/7 was ATRXloss, 4/7 were also p*TERT*mt (regarding the VAF, 2 were subclonal TMM activation, 1 non available, 1 same *ATRX-pTERT* VAF) and 83% (6/7) of MAPK activated gliomas also exhibited ALT + status (4/5 with ATRXloss, all p*TERT*wt). Importantly, 19% of *ATRX*-altered samples identified by NGS in our database were GBMs, of which 42% (8/19) were ALT + . This finding is noteworthy as the subcellular mechanisms and molecular alterations differ between ALT and telomerase contexts, impacting clinical behavior and tumor evolution [21, 27]. Hereby, the tendency toward a better prognosis for ALT + cases (Fig. 3d) should be interpreted in light of tumor type (Supplementary Fig. 1 ). Interestingly, the *p* value (though not significant) was better using TMM (*p* = 0.15) than IHC (*p* = 0.36) for survival prediction.

### Functional testing identifies pathogenic missense *ATRX* mutations

Comparing immunohistochemical detection of ATRX loss with TMM status, 92 of 115 samples (80%) showed concordance: 57 were ATRXloss/ALT + and 35 were TA + /ATRXwt. However, three samples with ATRX loss by IHC were classified as TA + by functional testing (possible false positives for IHC). These included a glioneural tumor with a heterozygous alteration, a GBM with a clonal alteration, and a histone-mutant tumor without sequencing data, possibly reflecting secondary hits without biological significance. Additionally, 20 samples were ALT + but showed wild-type ATRX by IHC (false negatives). This was expected for 10 samples with missense mutations, but surprising for 7 samples bearing frameshift or nonsense alterations.

Considering the type of alteration in relation to the detected TMM, 88% (56/64) of LOF alterations (frameshift and nonsense mutations) were classified as ALT + (Fig. 3c). Among the 8 samples with LOF alteration but TA + status, 5 were ATRXwt by IHC, 2 showed ATRXloss (a GBM with a clonal mutation and a glioneural with a heterozygous mutation), and 1 lacked IHC data. These observations may be linked to either secondary mutations without biological impact or minor subclonal alterations.

In sum, using NGS as the reference, the sensitivity and specificity for LOF mutation detection were 88% and 95% for TMM testing, and 80% and 100% for IHC, respectively. When including missense mutations, sensitivity remained stable for TMM testing (82%) but dropped to 65% for IHC, with most discrepancies related to missense mutations. Regarding the 28 missense alterations (Fig. 3c), 19 were detected as ALT + (8 of which showed ATRX loss by IHC and were further classified as LOF), while 9 were classified as TA + and preserved ATRX expression by IHC (8 GBM and 1 OD), considered likely secondary mutations (Supplementary Table 1).

**Fig. 4:**
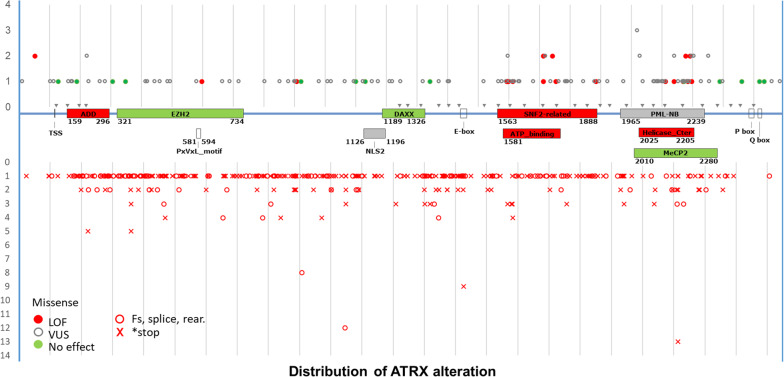
Distribution of *ATRX* Gene Alterations across ATRX Protein Domains. The ATRX coding sequence is represented in the center, with grey arrows indicating exon-exon junctions. Functional domains are highlighted in red: the ADD (ATRX-DNMT3-DNMT3L) domain, involved in binding H3K9me^3^ marks, and the two helicase domains (ATPase and C-terminal). Interaction domains are indicated in green for the following partners: EZH2 (enhancer of zeste homolog 2), HP1α (heterochromatin protein 1 alpha), MECP2 (methyl-CpG binding protein 2), and DAXX (death-domain associated protein). Localization signals, such as nuclear localization signals 1 and 2 (NLS1, NLS2) and PML nuclear bodies (PML-NB), are shown in grey. Above the gene, circles represent missense mutations classified as pathogenic (red), benign (green), or of unknown significance (grey). Below the gene, open red circles indicate frameshift, splice site mutation or rearrangement while red crosses indicate nonsense mutations. Abbreviations: VUS, variant of unknown significance; LOF, loss of function; Fs: frameshift

Another factor to be taken into account to classify a mutation as pathogenic is its impact on protein translation. Mutations from both databases were reported across all 36 exons of ATRX, with higher frequencies observed in the ADD and helicase domains. Frameshift and nonsense mutations (Fig. 4, lower track) were distributed randomly, with only 30% (153/504) located within functional domains. In contrast, 59% of missense mutations occurred within functional domains (ADD, SNF2-related, and helicase C-terminal). Interestingly, this proportion reached 83% (20/24) for mutations classified as LOF and dropped to 39% (5/13) for those deemed non-pathogenic. These findings further support our functional testing results.

In conclusion, the concordance of IHC with TMM was of 80% (92/115) but decreased to 63% (17/27) when focused specifically on samples with missense alterations. The sensitivity for missense mutation detection was of 82% for TMM and dropped to 65% for IHC. Importantly, 83% of mutations we classified as pathogenic occurred in functional domains, against 39% for non-pathogenic alterations. These results underscore the importance of functional TMM testing to improve diagnostic accuracy.

## Discussion

Numerous approaches have been proposed to determine the TMM in tumors. Beyond the reference standard functional assays such as C-circle detection, TeloFISH, and telomere Southern blot (Telo-Blot), recent studies have explored NGS-based modeling. For instance, TelomereHunter estimates telomere content (including telomeric variants) using WGS, as initially described by Feuerback [23], and later by Pickett et al. [24]. Other algorithms focus on identifying specific telomeric variant repeats (TVR) and interstitial telomeric sequences (ITSs), using truncating ATRX mutations as a surrogate gold standard for ALT positivity [26]. One recent study integrated TL and TVR distribution but also critically addressed the limitations of using truncating *ATRX* mutations as a reference. Using C-circle assays, it demonstrated that 27% of tumors with truncating variants were in fact ALT negative [25]. Importantly, none of these NGS-based models assessed missense mutations, as ATRX-altered samples were mostly used as ALT positive controls, and were typically limited to LOF variants. For instance, in the Pickett et al. study [24], eight of our cohort samples were included, all carrying LOF alterations (frameshift or nonsense mutations).

These approaches are useful for large-scale data analysis but remain inadequate for routine diagnostic use. Recently, long-read telomere sequencing has shown high accuracy in TMM classification and can simultaneously provide mutational information, making it a promising alternative, particularly for frozen samples [10]. However, the TeloDIAG offers several practical advantages: it is most cost-effective, can be completed within two days on FFPE samples, requires only a small amount of DNA (5 to 35 ng), and does not rely on complex bioinformatics.

### Diagnostic utility and limitations of ATRX testing in glioma

We report here that TMM phenotyping demonstrates higher sensitivity than ATRX IHC testing, particularly for missense alterations (82% vs. 65%) (this sensitivity is underestimated, as not all missense mutations are pathogenic). These finding has important diagnostic implications, given that the WHO 2021 classification recommends ATRX testing for the classification of IDH-mutant astrocytomas. In cases of false positives or negatives, the main surrogate marker used to distinguish IDH-mutant tumors is 1p/19q codeletion, which is required for oligodendroglioma (OD) classification. However, in samples with low tumor cellularity (below 20–30%), codeletions may be missed by comparative genomic hybridization (CGH) or by copy-number variation (CNV) analysis using NGS. Moreover, some atypical tumors may lack codeletion but still carry p*TERT* mutation (associated with OD) [21], or may show the opposite pattern [28]. Additionally, certain IDH–wild-type gliomas also harbor ATRX alterations [27]. We have previously shown that approximately 20% of p*TERT* wild-type glioblastomas (GBM) activate ALT, most without detectable ATRX loss by IHC, and that these tumors tend to have a better prognosis [21, 27]. This subgroup may represent a distinct biological entity with potential for targeted therapeutic strategies in the future.

### Expanded horizons for ALT activation in brain tumors and emerging therapeutic options

Unexpectedly, a significant proportion of ATRX-altered gliomas (18% in the TCGA cohort and 28% in our local cohort) were not classic IDH-mutant astrocytomas or histone-mutant gliomas. Instead, *ATRX* alterations were also found in GBMs (13–19%), ODs (2%), and tumors of glioneural origin with MAPK pathway alterations, including NF1, BRAF, and FGFR1 (2–7%). Using the TeloDIAG assay, we confirmed that 8 out of 19 GBM samples with *ATRX* alterations were indeed ALT + . This finding is consistent with our previous observation that approximately 20% of pTERTwt GBM are ALT + [21]. These results expand the landscape of *ATRX* alterations beyond the traditionally recognized tumor types. Importantly, GBMs with ATRX loss have been associated with better clinical outcome [27], a trend we also observed in ALT + GBMs, regardless of *ATRX* status [21]. Here, we also reported a stronger prognostic discrimination with TMM compared to ATRX testing, although this conclusion is limited by the heterogeneity of the diagnostic cohort. This opens new avenues for patient stratification and highlights the potential clinical relevance of TMM determination.

Among MAPK pathway-altered tumors, 6 out of 7 cases were ultimately classified as ALT + . We and others have previously reported that specific MAPK pathway mutations (BRAF, FGFR1, and NF1) can promote activation of the ALT mechanism to maintain telomere length [29, 30]. In pediatric high-grade gliomas (pHGG), without IDH or p*TERT* mutations, Telo-FISH analysis identified ALT activity in 24 out of 52 tumors, 9 of which did not harbor histone mutations [31]. More recently, a study using the CC assay combined with TL measurement confirmed ALT positivity in 38% (24 out of 63) of pHGG, including 40 non-histone-mutant tumors. Notably, 5 of these exhibited mismatch repair deficiency (dMMR), and 3 displayed a high tumor mutation burden (TMB) [32]. This is particularly relevant since high TMB and dMMR have also been identified in adult GBMs with ATRX loss [33]. Such molecular features are associated with enhanced immunogenicity and may identify patients who are more likely to benefit from immunotherapy. Supporting this, Hariharan et al. reported that ALT + IDH-mutant astrocytomas exhibit a more active innate immune response compared to their ATRX wild-type telomerase-positive OD counterparts [34]. These findings further underline the biological distinctiveness of ALT + tumors and suggest a potential link between TMM, DNA damage response and immunotherapeutic responsiveness. Clues of this assumption will soon be available, thanks to the ongoing clinical evaluation of nivolumab in IDH-mutant astrocytoma.

In parallel, various therapeutic strategies are being explored to specifically target the ALT pathway. These include recombination inhibitors, PARP-inhibitor, G-quadruplex stabilizers, and ALT-associated PML body (APB) inhibitors [35–38]. As an example, in vitro*,* ALT + H3.3 mutant have higher DNA damage and better respond to PARP inhibitor than their TA + counterpart [37]. Similarly, targeting ATR in ALT-associated HRD (homologous recombination defect) tumors has produced remarkable responses in metastatic melanoma [39]. Ongoing clinical trials targeting HRD in A and GBM (NCT03991832, NCT05188508) will be informative when interpreted in relation to TMM status. Given the aggressive nature and poor prognosis of both adult and pediatric high-grade gliomas (HGG), identifying ALT activation through functional testing could have dual value, serving not only as diagnostic tool but also as theranostic marker, guiding patients toward emerging ALT-targeted therapies.

### TeloDIAG: a simple and universal test for ALT + tumors

Using the TeloDIAG assay, we identified 20 ALT + samples among 35 tumors bearing *ATRX* alteration and classified as ATRXwt by IHC, representing false negatives. Similarly, Stundon et al. reported a 50% false-negative rate (24 cases) in pediatric HGG when using the CC assay combined with telomere sequencing [32], a finding corroborated by Minasi et al. [31]. These discrepancies may be attributed to technical limitations and the challenges of interpreting IHC results, particularly in the presence of missense mutations that preserve ATRX protein expression despite functional loss. In addition, mutations that activate the ALT pathway independently of ATRX also contribute to ALT + phenotypes. Diplas et al. identified *SMARCAL1* as a novel ALT activator in gliomas lacking both *pTERT* and *ATRX* mutations [40]. In sarcomas*, TOP3A* amplification is a known driver of ALT in *ATRX* wild-type tumors [41], and this alteration has also been observed in pediatric HGG [42]. In neuroendocrine pancreatic tumors, DAXX, the histone chaperone partner of ATRX, is frequently altered in ALT + cases, and has similarly been implicated in pHGG [15]. The spectrum of ALT activators continues to expand, likely varying with glioma subtype and etiology. As new functional testing technologies for ALT become available, identifying these alternative drivers and their biological context is critical for the development of targeted therapies. In this context, functional testing is the most accurate strategy for determining the TMM status, offering a reliable basis for both diagnosis and therapeutic-decision making.

## Conclusion

This study highlights the limitations of ATRX genetic sequencing and IHC assessment for clinical diagnosis, and underscores the value of functional TMM testing, such as TeloDIAG, in accurately identifying ALT + tumors. We provide proof of concept that a simple, robust assay like TeloDIAG can support the development of a curated database to assess the pathogenicity of *ATRX* alterations. Beyond its diagnostic value, such functional testing could rapidly become a companion biomarker in future clinical trials targeting telomere biology, paving the way for personalized therapeutic strategies in this era of precision oncology.

## Supplementary Information

Below is the link to the electronic supplementary material.

Supplementary Material 1. Summary table of ATRX alterations identified in the two datasets, annotated with their pathological significance.

Supplementary Material 2. Patients (N=105) were classified in two groups as a function of tumors grade : High (GBM and H3.3mt), low (OD, A, MAPK), and sub-classified in regard to the IHC or TMM testing results. The median survival is indicated. OD, oligodendroglioma; A, Astrocyotoma IDHmt; GBM, Glioblastoma; H3.3mt, histone mutant; MAPK, tumors with MAPK pathway alteration; g, grade; IHC, immunohistochemistry; TMM, telomere maintenance mechanism.

## Data Availability

No datasets were generated during the current study. Part of datas was extracted from the TCGA database (see Material and Method section), including the EXTEND signature.

## Abbreviations

ALT: Alternative lengthening of telomere
AMP: Amplification
ATRX: Ataxia alpha thalassemia/mental retardation syndrome X-linked
CC: C-circle
CGH: Comparative genomic hybridization
CNV: Copy number variation
DELINS: Deletion and/or insertion
Fs: Frameshift
GBM: Glioblastoma
H3.3mt: Histone H3.3 mutant glioma
HOMO DEL: Homozygous deletion
IHC: Immune-histo-chemistry
LOF: Loss-of-function
MAPK mutant: Glioma with NF1, BRAF or FGFR1 mutations
OD: Oligodendroglioma
na: Not available
NGS: Next generation sequencing
ni: Non informative
TA: Telomerase activity
TC: Telomere content
TL: Telomere length
TMM: Telomere maintenance mechnism
VUS: Variant of unknown significance
