# Supplementary material for: Functional analysis of telomere maintenance mechanisms is more informative than immunohistochemistry for ATRX mutation interpretation in Gliomas
Source: Acta Neuropathol Commun. 2025 Dec 20;14:25. doi: 10.1186/s40478-025-02164-z (PMC12831451; doi:10.1186/s40478-025-02164-z)
Supplement: Supplementary file 2 — Supplementary Material 2. Patients (N=105) were classified in two groups as a function of tumors grade : High (GBM and H3.3mt), low (OD, A, MAPK), and sub-classified in regard to the IHC or TMM testing results. The median survival is indicated. OD, oligodendroglioma; A, Astrocyotoma IDHmt; GBM, Glioblastoma; H3.3mt, histone mutant; MAPK, tumors with MAPK pathway alteration; g, grade; IHC, immunohistochemistry; TMM, telomere maintenance mechanism. [file 40478_2025_2164_MOESM2_ESM.pptx]

## Slide 1
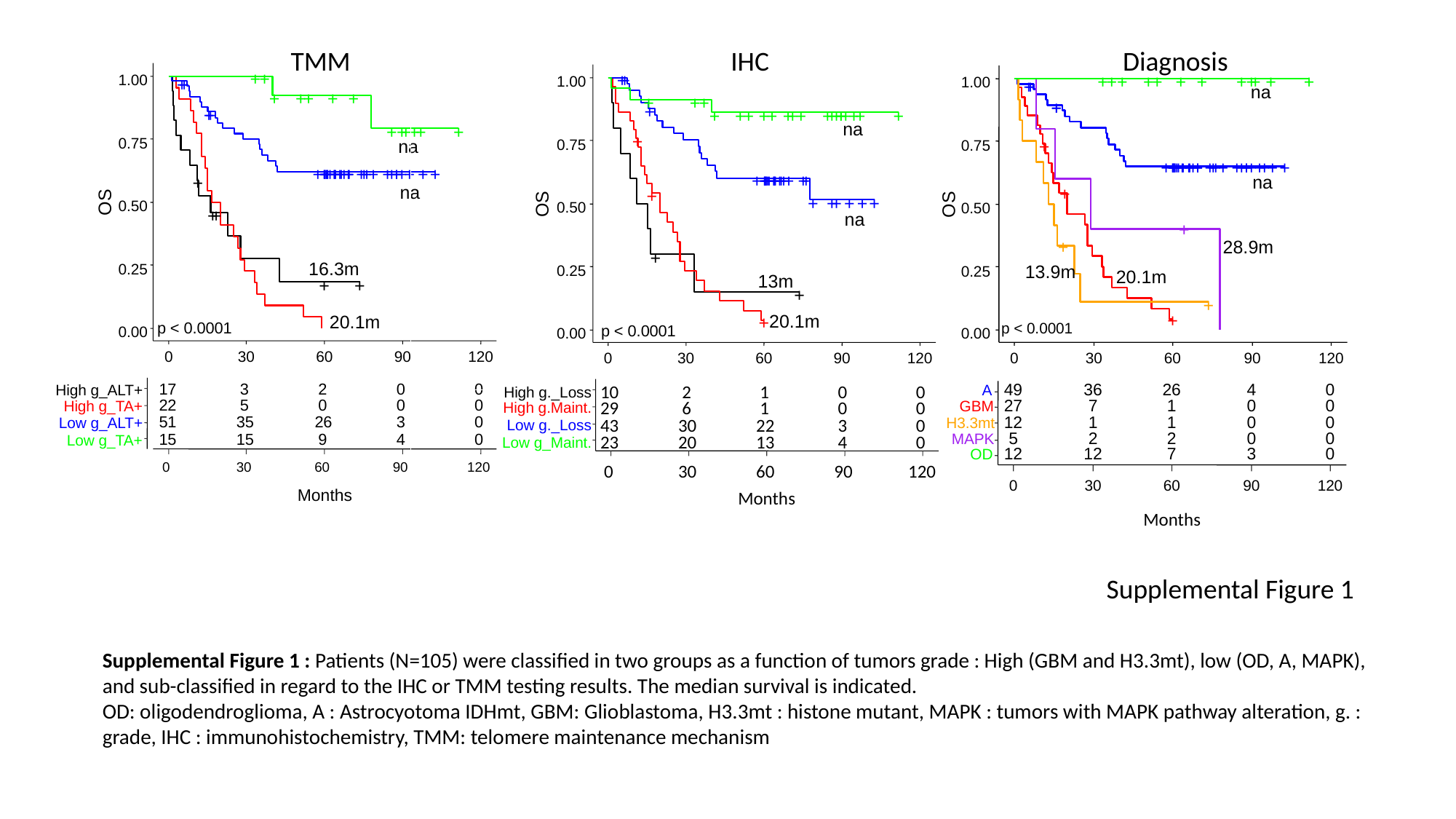

+
+
1.00
+
+
+
+
+
+
+
+
+
+
+
+
+
+
+
+
+
+
+
+
+
+
0.75
+
+
+
+
+
+
+
+
+
+
+
+
+
+
+
+
+
+
+
+
+
+
+
0.50
+
0.25
+
+
p < 0.0001
0.00
0
30
60
90
120
10
2
1
0
0
29
6
1
0
0
43
30
22
3
0
23
20
13
4
0
0
30
60
90
120
Months
High g._Loss
High g.Maint.
Low g._Loss
Low g_Maint.
OS
TMM
+
+
1.00
+
+
+
+
+
+
+
+
+
+
+
+
+
+
+
0.75
+
+
+
+
+
+
+
+
+
+
+
+
+
+
+
+
+
+
+
+
+
+
+
+
+
+
+
+
+
OS
0.50
+
+
0.25
+
+
p < 0.0001
0.00
0
30
60
90
120
17
3
2
0
0
High g_ALT+
22
5
0
0
0
High g_TA+
51
35
26
3
0
Low g_ALT+
15
15
9
4
0
Low g_TA+
0
30
60
90
120
Months
na
na
16.3m
20.1m
Diagnosis
IHC
+
+
+
+
+
+
+
+
+
+
+
+
1.00
na
+
+
+
+
+
0.75
+
+
+
+
+
+
+
+
+
+
+
+
+
+
+
+
+
+
+
+
+
+
+
+
+
+
+
+
na
+
OS
0.50
+
+
0.25
+
+
p < 0.0001
0.00
0
30
60
90
120
49
36
26
4
0
A
27
7
1
0
0
GBM
12
1
1
0
0
H3.3mt
5
2
2
0
0
MAPK
12
12
7
3
0
OD
0
30
60
90
120
Months
na
na
28.9m
13.9m
20.1m
13m
20.1m
Supplemental Figure 1
Supplemental Figure 1 : Patients (N=105) were classified in two groups as a function of tumors grade : High (GBM and H3.3mt), low (OD, A, MAPK), and sub-classified in regard to the IHC or TMM testing results. The median survival is indicated.
OD: oligodendroglioma, A : Astrocyotoma IDHmt, GBM: Glioblastoma, H3.3mt : histone mutant, MAPK : tumors with MAPK pathway alteration, g. : grade, IHC : immunohistochemistry, TMM: telomere maintenance mechanism
